# Assessment of the Accuracy of Nutrition Label and Chemical Composition of Plant-Based Milks Available on the Italian Market

**DOI:** 10.3390/foods12173207

**Published:** 2023-08-25

**Authors:** Vincenzo Lo Turco, Benedetta Sgrò, Ambrogina Albergamo, Vincenzo Nava, Rossana Rando, Angela Giorgia Potortì, Giuseppa Di Bella

**Affiliations:** 1 Department of Biomedical, Dental, Morphological and Functional Images Sciences (BIOMORF), University of Messina, Viale Annunziata, 98122 Messina, Italy; vloturco@unime.it (V.L.T.); benedetta.sgro@studenti.unime.it (B.S.); rrando@unime.it (R.R.); angela.potorti@unime.it (A.G.P.); gdibella@unime.it (G.D.B.); 2 Department of Veterinary Science, University of Messina, Viale Annunziata, 98168 Messina, Italy; vnava@unime.it

**Keywords:** plant-based milks, milk alternatives, nutrition labels, chemical composition, dietary reference intake, FA profile, tocopherols, total polyphenols, inorganic elements

## Abstract

Growing health, environmental, and ethical concerns have encouraged interest in plant-based milks (PBMs), but it remains questionable whether the nutrition labeling of these products is adequately reliable for consumers, and whether nutritional standards can be defined for a given PBM type. On this basis, cereal, pseudocereal, nut, and legume PBMs available on the Italian market were analyzed in order to check the accuracy of nutritional labels on packages and generate new or updated compositional data. Most labels provided inaccurate information, especially with respect to the declared energy, fat, and saturated fat. Cereal- and pseudocereal-based PBMs were generally characterized by high MUFA (34.04–59.35%) and PUFA (21.61–52.27%). Almond, soy, rice, and hazelnut beverages displayed the highest levels of total tocopherols (11.29–13.68 mg/L), while buckwheat and spelt PBMs had the highest total polyphenol content (34.25–52.27 mg GAE/100 mL). Major and trace elements greatly varied among samples, being more abundant in buckwheat and coconut-based drinks. A PCA confirmed that nutritional standards cannot be unequivocally established for a given PBM, and indicated that, among the investigated variables, inorganic elements had more weight in the sample differentiation. Overall, to reliably guide consumers in their dietary choices, there is a need for greater accuracy in the development of nutrition labels for PBMs, as well as greater effort in assessing the nutritional quality of the ever-increasing variety of products available on the market.

## 1. Introduction

Countless human and non-human lives and associated ecologies have been entangled for millennia in dairy milk production and consumption, while creating certain social and ecological vulnerabilities that ultimately are casting clouds over the future of the sector. In fact, on the one hand, conventional cow milk has been introduced especially in Western diets as a staple food due to its high calcium and vitamin D contents, which are beneficial for bone health [1]; on the other hand, concerns regarding the safety of such a product (e.g., lactose intolerance and cow milk protein allergy, high saturated fat and sugar levels, hormone and antibiotic use in cattle) and the environmental impact of the sector (e.g., loss of landscapes and biodiversity, soil degradation, and air and water pollution) are currently leading the global milk industry through a significant transition [2].

This is inevitably reflected in the latest numbers provided by the dairy market and its projections. In 2022, world milk production, for example, was forecast at around 930 million tons, with a +0.6% from 2021. This was principally driven by volume expansions in Asia, but, at the same time, offset by a sizable decline in Europe, South America, Oceania, and Africa, as well as a flattened production in North America [3]. With a focus on the European Union (EU)—the second largest global milk producer—the production is projected to grow in the future more slowly than the world average (~0.5%), mainly because EU countries are gradually taking action towards policies embracing more sustainable food production and consumption models (e.g., the Farm to Fork Strategy of the European Green Deal) [4].

In this scenario, dairy alternatives, especially in the form of plant-based milks (PBMs), are emerging as fundamental replacements to conventional milk in many regions of the northern hemisphere, such as the EU, North America, and East Asia. The global market of PBMs amounted to USD 27.3 billion in 2022, and it is forecast to reach USD 44 billion by the end of 2027, with a compound annual growth rate (CARG) of 10.4% [5]. In Italy, the market for PBMs was already growing in 2016, and, while soy drink grew by +2% in popularity, PBMs based on almond, hazelnut, oats, and coconut increased by +75%, despite having a ~30% higher price than soy drinks [6].

Key drivers of the rising interest in plant-based replacements for dairy products are (i) consumers’ environmental and health concerns about cow milk; (ii) the now more conscious dietary lifestyles such as vegetarianism, veganism, and flexitarianism [7]; and (iii) the marketed health properties of these products [8].

Although soy, almond, and coconut milks have played an indisputable role in the history of human nutrition, more than twenty PBMs are commercially available today, made from nuts (e.g., cashews, hazelnuts), grains (e.g., rice, oat), legumes (e.g., soy and peas), seeds (flax, hemp, sesame, chia), and fruits (banana) [9].

To increase consumer acceptance, PBMs are typically designed to look, feel, taste, and nutritionally resemble cow milk [10]. In practice, however, every type of PBM has its own organoleptic, technological, and nutritional profiles that mainly depend on the plant source and the production process.

In general terms, PBMs may be produced by (i) the wet process, based on soaking and wet milling of the raw material, and subsequent separation of solids by centrifugation or filtration to obtain smooth and milky final textures; or (ii) the dry process, involving the dry milling of raw materials and the following extraction of the flour in water [11]. During the final formulation, the beverage may be added with sugars, flavors, and additives (i.e., gelling agents), as well as fortified by micronutrients that are generally lacking in plant ingredients (e.g., protein, B_12_ and D vitamins, and minerals such as Ca and I) [12]. Based on their production process, PBMs have a lower environmental impact than dairy milk in terms of carbon footprint, water consumption, land use, and potential ecosystem damage [13].

Compared with the cow milk, PBMs are characterized by the presence of health-promoting compounds, such as dietary fiber, phytosterols, and antioxidants (i.e., tocopherols, phenolics, and isoflavonoids). Additionally, they have lower carbohydrate contents if they have no added sugars, and lower fat—mainly mono- and polyunsaturated fatty acids (MUFAs and PUFAs) [14,15]. However, PBMs suffer from a low bioavailability of these nutrients due to the presence of anti-nutrients, such as saponins, oxalates, and phytates [7], which, fortunately, may be reduced or even removed through an extended mechanical and thermal pre-processing of the raw material (e.g., roasting, dehulling, blanching, soaking, cooking, and sprouting) [8]. PBMs also lack a significant protein portion, B vitamins, and Ca, although they have comparable contents of other minerals, such as potassium, magnesium, and phosphorus [16].

Overall, recent literature has focused on the most common soy, almond, rice, and coconut PBMs, showing that their chemical composition greatly differs from beverage to beverage and even varies within the same type of beverage, mainly depending on the plant source, product processing, and formulation [7,16,17]. Considering the ever-growing market, reliable nutritional standards for PBMs are highly desired not only to guide production and innovation, but also to develop accurate nutrition facts labels and nutrition/health claims that are useful in helping consumers to make better-informed choices [18]. Existing evidence shows that both nutrition facts and claims are attractive for consumers and can effectively affect food choices and promote healthier diets, at least in some population groups [19,20]. However, nutrition facts labels are often not very accurate, and nutrition/health claims are still found for products of lower nutritional quality, which can mislead consumers into consuming a higher volume of products with an apparently “better” nutritional quality [21,22]. In the PBM market, the accuracy of their information is critical, since it has an impact on efforts to reduce dietary fat or sodium, as well as to ensure an adequate intake of minerals or vitamins.

Within this background, the aim of the study was to systematically assess the chemical composition and nutritional quality of different PBMs available on the Italian market by (i) verifying the matching of nutritional labels and potential nutrition/health claims; (ii) producing updated or new compositional data, with particular emphasis on micronutrients; and (iii) studying the nutritional intake of such beverages through dietary modelling in male and female adults, in line with the Regulation (EU) No. 1169/2011 [23]. To the best knowledge of the authors, this is the first study aiming to experimentally assess the chemical composition and nutritional quality of an array of PBMs available on the Italian market. In fact, only Angelino et al. [6] examined the overall quality of PBMs from the Italian market base, however, only on the data of nutrition labels reported on packaging.

## 2. Materials and Methods

### 2.1. Samples

During the period of January–April 2023, 12 types of unflavored and organic PBMs belonging to 7 different commercial brands marketed throughout Italy were selected from major supermarkets (i.e., Carrefour, Conad, Despar, Pam Panorama, Coop, and Sidis) in the province of Messina (Sicily, Italy). The PBMs were based on nuts (i.e., coconut, almond, and walnut), cereals (i.e., rice, oats, sorghum, millet, and spelt), pseudocereals (i.e., buckwheat), and legumes (i.e., soy), as well as mixed plant ingredients (i.e., rice/coconut and rice/hazelnut), and most of them included formulation salt and other fat (i.e., sunflower oil) and/or carbohydrate (i.e., rice starch and syrup) sources. To reach such a product selection, specific criteria were considered: (i) the PBM must be organic and unflavored; (ii) the PBM must have no more than two plant ingredients; and (iii) the PBM must be available at least in one of the selected supermarkets.

Each PBM was purchased in five replicates of the same brand, but different lots, at different supermarkets and times during the study period; a total of n = 60 PBMs were collected. Table 1 provides an overview of all PBMs used for the study, along with related information, such as the list of ingredients, nutrition facts label, and nutrition claims as defined by Reg. (EU) 1169/2011 [23] and Reg. (EC) 1924/2006 [24].

All samples were kept in their original packages and transferred to the laboratory in an ice box. Hence, around 600 mL of each PBM was lyophilized by a CHRIST freeze-dryer (model D-37520 Osterode am Harz, Germany) to obtain ~80 g of dry mass from every product. All PBMs were stored at −18 °C until analysis and were analyzed before their expiry date.

### 2.2. Chemicals and Reagents

Solvents (i.e., chloroform, methanol, n-hexane, n-heptane and ethyl acetate) were UHPLC/MS-grade and purchased from Merck (Darmstadt, Germany). Reagents such as HNO_3_ (65% *v*/*v*) and H_2_O_2_ (30% *v*/*v*) were of Suprapur grade (Mallinckrodt Baker, Milan, Italy), and HCl (37%) was obtained from Merck (Darmstadt, Germany). Ultrapure water (<5 mg/L TOC) was obtained from a Barnstead Smart2Pure 12 water purification system (Thermo Scientific, Milan, Italy).

Reference standard solutions of fatty acid methyl esters (FAMEs, C4–C24), tocopherols (α-tocopherol, γ-tocopherol and δ-tocopherol), gallic acid, and the Folin–Ciocalteu reagent were obtained from Sigma-Aldrich (Darmstadt, Germany). Stock standard solutions of inorganic elements, such as Na, Mg, K, Ca, Mn, Fe, Cu, Zn, Se, Ni, Cr, As, Cd, and Pb (1000 mg/L in 2% HNO_3_, each); on-line internal standard solutions of ^45^Sc, ^73^Ge, ^115^In, and ^209^Bi (1000 mg/L in 2% HNO_3_) to correct instrumental drift and matrix deviation; and the internal standard solution of Re (1000 mg/L in 2% HNO_3_) were all obtained by Fluka (Milan, Italy).

For element analysis, all laboratory equipment was washed with 5% HNO_3_ before use to avoid undesirable metal contamination.

### 2.3. Proximate Composition

The proximate composition of every type of PBM was evaluated according to the AOAC official protocols of analysis [25]. Dietary fiber was determined through the Megazyme assay kit (International Ireland Ltd., Wicklow, Ireland) in accordance with the AOAC Official Method 991.43 and the AACC Method 32-07.01. Specifically, two sample aliquots (1 g each) were treated in parallel by α-amylase at 80 °C and then digested with protease and amyloglucosidase at 60 °C. Subsequently, the solutions were cooled at ~40 °C and mixed with ethanol to precipitate fiber. The fiber residues were filtered, washed with organic solvents, and dried, and the mean weight was calculated.

At this stage, one residue was incubated at 500 °C until a constant weight was achieved (~12 h) to determine ash, whereas the other residue was analyzed for crude protein by the AOAC Official Method 976.05. In this case, digestion of the residue was performed with H_2_SO_4_, CuSeO_3_ • 2H_2_O, and K_2_SO_4_ by the SpeedDigester K-439 (Büchi, Switzerland), and then analyzed by the KjelMaster System K-375 (Büchi, Switzerland). The resulting solution was treated with NaOH to develop NH_3_, and the nitrogen content was evaluated by titration with HCl. Then, the calculation of crude protein (%) was carried out by multiplying the nitrogen (%) by a conversion factor of 6.25. Hence, dietary fiber was equal to the mean weight of the dried residue, minus the weight of protein and ash.

The total fat was determined according to the AOAC method 920.39. Briefly, ~15 g of dried PBM was extracted using a Soxhlet apparatus with n-heptane for 6 h. Then, the extract was dried with a rotating evaporator (Heidolph Instruments GmbH and Co., Schwabach, Germany) and the extraction yield was gravimetrically determined.

### 2.4. FA Profile

For the elucidation of the FA composition of every PBM, the lipid extract was recovered through the addition of 1 mL of methanolic KOH (2M), and transmethylated at 70 °C for 2 min to obtain fatty acid methyl esters (FAMEs) [26]. Then, the supernatant was diluted to 2:1 with heptane and analyzed by a gas chromatography system equipped with a split/splitless injector and a flame ionization detector (GC-FID, Dani Master GC, Dani Instrument, Milan, Italy) according to the operating conditions already reported by Di Bella et al. [27] and Albergamo and colleagues [28]. A Zebron ZB-WAX capillary column (length 30 m, internal diameter 0.25 mm, film thickness 0.25 μm, Phenomenex, Torrance, CA, USA) was used along with the carrier gas (He) at a constant linear velocity of 29 cm/s. The column oven temperature program increased from 130 °C to 200 °C at 3 °C/min. The injector and the flame ionization detector temperatures were set to 220 °C and 240 °C, respectively. An injection volume of 1 µL with a split ratio of 50:1 was employed. The Clarity Chromatography v. 4.0.2 software was considered for data acquisition and management. FAMEs were identified by comparing the reference retention times of the compounds present in the standard mix. Individual FAMEs (%) were calculated with respect to the total chromatogram area. Triplicate measurements for each sample were carried out.

The nutritional quality of the different FA compositions in relation to cardiovascular and metabolic health was determined by calculating several indices, such as the atherogenicity index (AI), the thrombogenicity index (TI) and hypocholesterolemic/hypercholesterolemic ratio (h/H), as already reported in our previous studies [28,29,30]:

$$AI=\frac{C12+4\left(C14\right)+C16}{\sum MUFA+\sum PUFA\;n-6+\sum PUFA\;n-3}$$

$$TI=\frac{C14+C16+C18}{0.5\left(\sum MUFA\right)+0.5\left(\sum PUFA\;n-6\right)+3\left(\sum PUFA\;n-3\right)+\frac{\sum PUFA\;n-3}{\sum PUFA\;n-6}}$$

hH=C18:1+C18:2+C18:3C12:0+C14:0+C16:0

Another important determinant of health, namely, the n-6/n-3 ratio, was also evaluated.

### 2.5. Tocopherols

For the extraction of tocopherols, 2 g of each freeze-dried PBM was mixed with 4 mL of n-hexane in a dark tube, stirred, and added with further 2 mL of methanolic KOH. Then, the mixture was stirred and centrifuged for 10 min at 4000× *g* at 4 °C. The supernatant was filtered through a 0.2 μm nylon filter, transferred into an amber vial, and analyzed by high-performance liquid chromatography coupled to a fluorescence detector (HPLC-FD) (Shimadzu, Milan, Italy) according to a protocol already proposed by Lo Turco and colleagues [31]. A LiChrosorb Si 60 (5 µm) column (4.6 mm I.D. × 250 mm, Merck, Darmstadt, Germany) protected by a LiChroCART 4-4 guard column with the same stationary phase (Merck, Darmstadt, Germany) was employed. A solution of n-hexane/ethyl acetate (90:10 *v*/*v*) was used as the mobile phase. The injection volume was 20 µL. The analyses were carried out at 40 °C in isocratic conditions with a flow rate of 0.8 mL/min. The fluorescence excitation and emission wavelengths were 295 nm and 330 nm, respectively. LabSolutions software ver. 5.10.153 (Shimadzu) was used for data acquisition and management. The tocopherols were identified by comparing the reference retention times of the commercial standards of α-, γ-, and δ-tocopherol. The quantification was carried out by the external calibration method, exploiting six-point calibration curves for each tocopherol. The total tocopherol content was calculated as the sum of the individual tocopherols. All determinations were carried out in triplicate.

### 2.6. Total Polyphenols

The total polyphenol content of PBMs was evaluated by following the method recently proposed by Albergamo and coworkers [28].

Briefly, 1.5 g of each freeze-dried PBM was added with 4 mL of chloroform–methanol solution (80:20, *v*/*v*). After stirring, the sample was centrifuged for 15 min at 4000× *g* (+4 °C) and the supernatant was filtered through a 0.45 μm nylon filter. Then, 10 µL of the supernatant was mixed with 240 μL of distilled water and 250 μL of Folin–Ciocalteau reagent, then added with 2.5 mL of Na_2_CO_3_ (7%). The sample was incubated in the dark at room temperature for 90 min, then analyzed with the UV–VIS spectrophotometer (UV-2401 PC, Shimadzu, Milan, Italy) at an absorbance wavelength of 760 nm. The quantification of total polyphenols was performed by constructing a suitable six-point calibration curve of gallic acid, and was expressed in terms of mg of gallic acid equivalent per 100 mL of product (mg GAE/100 mL). Triplicate measurements were conducted for every PBM.

### 2.7. Inorganic Elements

PBMs were mineralized by following the procedure already described by Crupi and colleagues [32]. Briefly, 0.5 g of each freeze-dried PBM was added to 8 mL of HNO_3_, 2 mL of H_2_O_2_, and 1 mL of the internal standard Re (0.5 mg/L) in PTFE (polytetrafluoroethylene) vessels. A microwave ETHOS 1 digestion system (Milestone, Bergamo, Italy) was employed for the mineralization of the sample with the following instrumental parameters: 10 min from 0 °C to 200 °C, then 20 min held at 200 °C, with a microwave power of 1000 W. After cooling down to room temperature, the extracts were properly diluted with ultrapure water and filtered before the ICP-MS analysis.

The determination of inorganic elements was performed using a quadrupole ICP-MS, iCAP-Q (Thermo Scientific, Waltham, MA, USA), equipped with an ASX-520 autosampler (Cetac Technologies Inc., Omaha, NE, USA) and provided with the Qtegra Intelligent Scientific Data Solution software (Thermo Scientific) for data acquisition and management.

The ICP-MS operating conditions already adopted in our previous works [33,34] were: RF power of 1550 W; sample depth and sample introduction flow rate of 5 mm and 0.93 mL/min, respectively; the flow rates of plasma, auxiliary, and carrier gases (Ar) were, respectively, 14 L/min, 0.8 L/min, and 1.1 L/min; the collision gas (He) flow rate was 4.7 mL/min; the dwell time was 1 s; the extract lens 1 voltage was 1.5 V; the spray chamber temperature was 2.7 °C; and the nebulizer pump was 0.1 rps. The integration times were 0.5 s/point for Fe, Se, and As and 0.1 s/point for the other elements. To integrate the peaks, 3 points for each mass and 3 replicate acquisitions were taken.

An external calibration procedure, based on the construction of six-point calibration plots with internal standard normalization, was adopted for quantitative purposes. All samples were analyzed in triplicate, along with analytical blanks.

### 2.8. Statistical Analysis

The SPSS 13.0 software package for Windows (SPSS Inc., Chicago, IL, USA) was used for the statistical analysis. Statistical analysis was performed using an initial multivariate matrix where the cases were the 60 PBM samples studied and the variables were the values of all experimental data. Caprylic, capric, and lauric acids, as well as Cr and Cd, were not included in the data set because their concentrations were below the LOQ in more than 60% of the total samples analyzed. The concentrations of As, Se, and Pb were below the LOQ in only a few samples, so a value of LOD/2 was assigned to the concentrations below the LOQ [35].

Based on the type of PBM, the dataset was divided into 12 groups. First, to highlight the differences between the groups, the Kruskal–Wallis one-way analysis of variance (ANOVA) was applied. Then, the data set was normalized to obtain equal importance for all variables [36], and a factor analysis with principal component extraction was performed to differentiate the samples from different PBMs according to the obtained experimental data, as has already been described in our previous works [37,38].

## 3. Results and Discussion

### 3.1. Accuracy of Nutrition Labels

The nutritional labels of PBMs experimentally determined are reported in Table 2.

It is recognized that it is not always possible for foods to contain the exact amount of nutrients labeled, owing to natural variations and variations arising from production and length of storage. However, it is important that the actual nutrient content of foods should not deviate substantially from labeled amounts, as the consumer could otherwise be misled. As a result, the European Commission has drawn up, in collaboration with EU Member States, guidance on the setting of tolerance for every nutrient value [39], i.e., the acceptable difference between the nutrient value declared on a label and that established during laboratory controls. The document set tolerances for the Reg. (EU) No. 1169/2011 on the provision of food information to consumers.

By comparing the nutrition facts labels reported on packaging with the nutrition labels experimentally determined in the laboratory, we found that the declared nutritional data of many PBMs were not very accurate (Table 1 and Table 2). Indeed, except for the declared fiber, which was always in line with experimental data, the declared Kcal and fat were out of the tolerance range in 83% of the analyzed products, being, in most cases, above the upper tolerance limit. The declared protein levels of most cereal (i.e., oat, spelt and millet), buckwheat, and soy PBMs were outside of the set tolerance. Similarly, buckwheat and all cereal PBMs, except the oat drink, had declared carbohydrates and related sugars below the lower tolerance limits (Table 1 and Table 2).

Around 67% of the selected PBMs presented nutrition claims in accordance with the Regulation (EC) no. 1924/2006, such as “with no added sugar”, “low fat”, and “source of calcium and vitamins” (Table 1).

The claim “with no added sugar” states that no sugar has been added to the PBM, nor does it contain added mono- or disaccharides or other foods used for their sweetening properties. The claim was always used appropriately with respect to the ingredient list of the PBM in which it appeared (i.e., rice, oat, sorghum, spelt, millet, coconut, and rice and coconut, Table 1).

The claim “low fat” appeared only in walnut and rice and coconut PBMs, as it can only be made when the product contains no more than 3 g of fat per 100 g for solids or 1.5 g of fat per 100 mL for liquids. According to the fat content, which was experimentally determined to be <1.5% (Table 2), both products correctly employed this claim.

The claim “source of calcium” was present only in walnut PBM, and it indicates that the product is a source of Ca when it contains at least a significant amount of this mineral as defined by the Annex I of Directive 90/496/EEC [40], namely, 15% of the recommended daily allowance (RDA) of Ca supplied by 100 g or 100 mL of product. The RDA of Ca is equal to 800 mg/day, and, according to the stated nutrition claim, 100 mL of walnut drink should provide 120 mg of Ca, which is thought to cover 15% of the RDA (Table 1). In the case of a value declared on a label that is equal to the minimum level specified in the conditions of use of the claim, the tolerance set for minerals and vitamins is:

$$Max.\;tolerance=rounding\;to\;3\;significant\;figures\;\left(upper\;bound\right)+45\%\;of\;the\;upper\;bound\;value$$

$$Min.\;tolerance=rounding\;to\;3\;significant\;figures\;\left(lower\;bound\right)-20\%\;of\;the\;lower\;bound\;value$$

Hence, maximum and minimum tolerance values of 174.6 mg/100 mL and 95.6 mg/100 mL can be defined for Ca. Based on the ICP-MS analysis of walnut PBM that will be later discusse, a content of Ca equal to 139.98 mg/100 mL and, consequently, within the accepted tolerance, was determined.

### 3.2. FA Profile

The FA compositions of different commercial PBMs are reported in Table 3. Based on the Kruskal-Wallis one-way ANOVA, almost all FAs, with exception of saturated decanoic and lauric acids, significantly varied among the investigated samples (*p* < 0.001).

Most PBMs were characterized by a lower content of saturated fatty acids (SFA, 10–15%), with palmitic acid being dominant, which showed its highest and lowest percentages, respectively, in rice and sorghum drinks (i.e., 15.89% and 6.58%). However, coconut and rice and coconut drinks contained more than 80% SFA due to shorter-chain FAs, such as caprylic, decanoic, lauric, and myristic acids, which amounted together to ~75%, thus reflecting the typical FA profile of *Cocos nucifera* L. [41,42].

MUFA ranged from ~6% (i.e., coconut and rice and coconut milks) to ~60% (i.e., almond and rice and hazelnut milks), and the FA most responsible for this variability was the oleic acid, which accordingly varied from ~6% to 59.48% in the same PBMs. Similarly, PUFA differed from 1–2% (i.e., coconut and rice and coconut drinks) to 57–63% (soy and walnut milks) due to the presence of linoleic acid, which, accordingly, represented 85–99% of the total PUFA in the investigated PBMs. The highest contents of oleic and linoleic acids, recorded in the almond, walnut, and rice and hazelnut PBMs, reflects the presence of the healthy nuts in PBMs, since almond and hazelnut are already well known for their very high contents of oleic acid, which is the main FA of the unsaturated fraction. On the other hand, walnut is notoriously characterized by the most abundant linoleic acid in its PUFA portion [43,44,45].

Interestingly, buckwheat, sorghum, spelt, and millet PBMs were characterized by similar FA profiles, probably because of the presence of cold-pressed sunflower oil in the formulation. Indeed, the FA distribution of these drinks differed from those of raw materials [46,47,48,49], thus being more similar to that of cold-pressed sunflower oil, which confirms that the drinks had very high contents of linoleic acid [50,51].

In the literature, the FA profiles of commercial PBMs were little explored. Additionally, some drinks, such as buckwheat, sorghum, spelt, and millet, have never been addressed before. Recently, Martínez-Padilla et al. [26] evaluated the FA profiles of some of the same drinks considered in this study (i.e., almond, coconut, oat, rice, and soy). Predictably, the comparison of results was quite troublesome, mainly due to the different percentages of the plant ingredients, as well as the potential presence of vegetable oils in the PBM formulation. However, the FA profiles of soy PBM resulted to be very similar to those of soybeans and soy milk produced on a laboratory scale using only soybeans and water, especially with respect to the most abundant FAs, such as linoleic (>50%), oleic (~20%), and palmitic (~10%) acids [52].

Indicators of the stimulation of cholesterol metabolism and platelet aggregation responsible for cardiovascular syndromes, such as the AI, TI, and h/H ratio, were calculated for all the beverages to evaluate the healthiness of their fat. These are listed in Table 3.

The PBMs showed generally low AI and TI indices—being directly proportional to SFAs—and, as a result, they demonstrated good potential for protection against coronary diseases. Specifically, AI was composed of between 0.082–0.091 (i.e., sorghum, millet, almond, and rice and hazelnut) and 0.24 (i.e., rice), while TI was in the range of 0.16 (i.e., rice and hazelnut) to 0.45 (i.e., rice). Comparable AIs and TIs were found in very healthy seed oils, such as linseed (respectively, 0.00 and 0.04), grapeseed (respectively, 0.08–0.09 and 0.24–0.26), hemp seed (respectively, 0.07 and 0.10), sesame (respectively, 0.13 and 0.26), and pomegranate seed (respectively, 0.42 and 0.75) [53,54,55,56].

However, coconut PBMs from this study showed greater AI (14.87–17.48) and TI (6.53–8.15) indices due to the higher SFA proportion.

The h/H values ranged from 0.11–0.12 (i.e., coconut and rice and coconut) to 12.49–12.55 (sorghum and rice and hazelnut). A higher level of this index—directly proportional to the PUFAs content—would be desirable, as it expresses the effect of FAs on cholesterol metabolism. In this respect, comparable h/H values were found in linseed (13.24) and grapeseed oils (11.07–12.28) [53,54].

Considering the n-6/n-3 ratio, walnut and soy beverages showed the most balanced ratios (respectively, 4.36 and 6.64). Other PBMs showed higher ratios, ranging between 10.25 (i.e., rice and coconut) and 55.81 (i.e., spelt). The literature has reported various examples of oily matrices with similar n-6/n-3 ratios, such as hempseed oil (3.79) on the one hand and sesame oil (50) on the other [54,55]. However, millet and buckwheat PBMs had very imbalanced ratios (respectively, 122.72 and 167.80) due to very high contents of linoleic acid and very small amounts of linolenic acid.

### 3.3. Tocopherols and Total Polyphenols

The contents of antioxidants, such as α-γ-δ-tocopherols, as well as their sums and total polyphenols, which were revealed in the PBMs are shown in Table 4. According to the Kruskal–Wallis one-way ANOVA, both the tocopherols and polyphenols were significantly different among the investigated PBMs (*p* < 0.001).

In almost every PBM, the concentration order was α- > γ- > δ-tocopherol, with the α-isomer varying between 0.55 mg/L and 12.30 mg/L and the γ- and δ-isomers in the ranges of 0.30–1.80 mg/L and 0.041–0.65 mg/L, respectively. The only exception was the soy drink, which showed higher levels of γ-and δ-tocopherols (8.67 mg/L and 2.85 mg/L) than the α- isomer (0.66 mg/L). As a result, the sums of tocopherols were found to be between 0.53 mg/L and 13.68 mg/L.

The almond drink presented the highest content of tocopherols (13.68 mg/L), followed by the soy and rice and hazelnuts PBMs (12.19 and 11.29 mg/L, respectively), while the coconut PBM had the lowest level (0.53 mg/L).

The highest total polyphenol content was detected in the buckwheat drink (52.27 mg GAE/100 mL), followed by the spelt (34.25 mg GAE/100 mL) and soy (20.61 mg GAE/100 mL) PBMs. Intermediate values were highlighted for most PBMs, such as rice, oat, sorghum, millet, almond, walnut, and rice and hazelnut (range: 13.16–18.01 mg GAE/100 mL). On the other hand, the lowest levels of total polyphenols were recorded in the rice and coconut and coconut drinks (respectively, 9.54 mg GAE/100 mL and 5.40 mg GAE/100 mL).

In this study, most of the selected PBMs were analyzed for tocopherols and total polyphenols for the first time. In fact, the literature has generally focused only on the most common soy, almond, and rice beverages, and highly variable contents of tocopherols and total polyphenols were generally outlined.

For example, Toro-Funes and colleagues focused on soy drinks obtained by ultra-high-pressure homogenization (UHPH) and confirmed that the γ-tocopherol was the most abundant isomer, followed by δ- and α-isoforms, although lower contents of total tocopherols were revealed (1.86–3.46 mg/L) [57]. The same authors also investigated almond drinks prepared by the conventional method and by UHPH. With respect to our study, they found a higher concentration of total tocopherols (50.63 mg/L) in the base product and much lower levels in UHPH drinks (3.15–9.51 mg/kg). In another study by Vanga and coworkers, the tocopherol content reported on the nutritional labels of commercial almond, soy, and rice drinks was equal to 3.84 mg/L, 4.00 mg/L, and 3.00 mg/L [15].

Moretto et al. reported that commercial oat and soy drinks, prepared only by grinding and soaking the plant ingredients in water, had levels of total polyphenols similar to those found in this study (respectively, 18.12 mg GAE/100 g and 19.26 mg GAE/100 g) [58].

Zahrani et al. reported lower polyphenol contents for soy and almond drinks prepared by the wet method (respectively, 5.17 mg/ 100 mL and 2.38 mg/100 mL) than those obtained in this study [59].

In a survey of commercial PBMs, Aly et al. revealed similar total polyphenols in coconut (8.10 mg GAE/100 g) and rice (12.41 mg GAE/100 g) drinks. However, soy, oat, and spelt showed non-comparable contents of such antioxidants [60].

Overall, PBMs differentiate from conventional milk in terms of the unique profiles of antioxidant compounds, such as tocopherols and polyphenols, although these may be impaired to varying degrees by processing [7]. However, such bioactive compounds are well known not only for improving the oxidative stability and shelf life of the beverage [58], but also for preventing cardiovascular, metabolic, and neurodegenerative diseases once taken up by the consumer [7].

### 3.4. Inorganic Elements

The Kruskal–Wallis one-way ANOVA test demonstrated that major elements, such as Na, K, Mg, and Ca; essential trace elements, such as Fe, Mn, Cu, Zn, Se, Ni, and Cr; and potentially toxic elements, such as As, Cd, and Pb, varied significantly among the selected PBMs, as reported in Table 5. In the selected PBMs, Na and K were the most abundant minerals (range: 237.51–2057.81 mg/L), followed by Ca (range: 119.44–1399.84 mg/L) and Mg (range: 53.09–278.26 mg/L). Based on the described ranges, the buckwheat drink was the PBM with the highest levels of K and Mg, and the walnut beverage stood out for its Ca content due to its functionalization with calcium phosphate (Table 1), in accordance with Regulations (EC) No. 1924/2006 and 1925/2006 [24,61]. The buckwheat, coconut, and almond PBMs were also characterized by high Ca levels (769.53–503.31 mg/L). However, the rice PBM was generally characterized by the lowest contents of such minerals. Considering essential trace elements, Fe and Zn were present at higher levels (respectively, 0.50–5.36 mg/L and 0.64–5.85 mg/L). The buckwheat, soy, and rice and coconut PBMs were generally characterized by the highest amounts of Fe (4.58–5.36 mg/L), Mn (1.80–2.94 mg/L), Cu (1.55–2.49 mg/L), and Zn (2.67–5.85 mg/L).

On the other hand, the rice drink had the lowest concentrations of Fe (0.50 mg/), Cu (0.08 mg/L), and Zn (0.64 mg/L), and the content of Se was <LOQ.

The contents of potentially toxic elements, such as As, Pb, and Cd, in PBMs resulted to be highly variable. Pb was determined in 10 products, with concentrations of 0.0051–0.017 mg/L; followed by As, found in 7 beverages in amounts between 0.0043–0.028 mg/L; and Cd was quantified in 3 PBMs in the range of 0.0042–0.013 mg/L. All of these elements were determined at high levels in buckwheat PBMs (As: 0.013 mg/L, Pb: 0.017 mg/L, and Cd: 0.013 mg/L), probably due the capacity of common buckwheat (*Fagopyrum esculentum* Moench) to develop mechanisms responsible for the increased uptake of toxic components without harming the plant organism, although the risk of toxicity in subsequent links of the food chain remains [62]. Additionally, the rice and rice and hazelnut PBMs were characterized by the highest levels of As due to the well-known capacity of the species *Oryza sativa* to take up such metalloids from the soil and water used for irrigation. Thus, this is a major reason for As being a dietary staple of half of the world’s population [63]. On the other hand, the spelt and millet PBMs were the least contaminated products, since they had all potentially toxic element <LOQs.

The European Union has set the maximum levels of Pb and Cd in different foodstuffs, but not in plant-based drinks [64]. However, the investigated PBMs did not exceed the maximum levels of Pb and Cd fixed for the raw materials from which they come, such as cereals and pseudocereals (respectively, 0.20 and 0.10 mg/kg), legumes (respectively, 0.10 and 0.020 mg/kg), and nuts (Cd: 0.20 mg/kg).

Most of the investigated PBMs were analyzed for their inorganic elements for the first time in this study. In fact, the literature has focused generally on soy, almond, coconut, and rice beverages, and has pointed out the highly variable profiles of inorganic elements due to the natural variability of plant ingredients, as well as the variation encountered in product formulation and processing [15,65,66].

However, Astolfi and colleagues evaluated inorganic elements in an array of popular and less common PBMs marketed in the province of Rome (Italy), and the profile of inorganic elements found in oat, soy, and walnut PBMs was comparable with that obtained in this previous study, most likely due to the analysis of products from the same brands [67].

### 3.5. PCA Analysis

As already discussed, the Kruskal–Wallis one-way ANOVA showed significant differences (*p* < 0.05) between samples from different PBMs for all experimental variables. Therefore, subsequent statistical analyses did not exclude any variables. First of all, the data eligibility for PCA was verified: the determinant value was 6.28 × 10^−40^ (i.e., above 0.00001, indicating the absence of multicollinearity); the Bartlett’s test of sphericity had a significance of 0.0001 (i.e., below 0.05, confirming that there was a patterned relationship among the variables); and the Kaiser–Meyer–Olkin measure (KMO) of sampling adequacy was 0.728 (i.e., above 0.6, showing the sampling adequacy).

**Figure 1:**
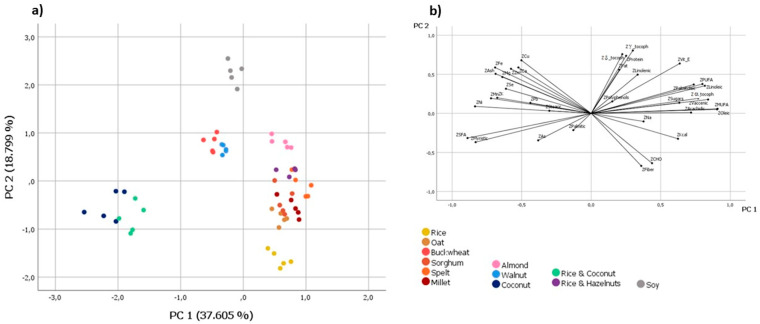
Score (**a**) and loading (**b**) plots of PC2 vs. PC1, with the samples categorized according to the type of PBM, obtained by considering all experimental data during statistical analysis.

Preliminary tests were followed by PCA. Seven principal components had eigenvalues greater than 1.0, thus satisfying the Kaiser criterion, and this accounted for a total variance of 89.090%. The PC1, which explained 37.605% of the total variation in the data, was strongly correlated with the FA profile. Specifically, it was positively correlated with MUFA (0.909), oleic acid (0.905), cis-vaccenic acid (C18:1 n-7) (0.841), linoleic acid (0.817), PUFA (0.800), and α-tocopherol, while it was significantly negatively correlated with SFA (−0.889) and myristic acid (−0.831). In addition, another significant negative correlation was observed with Ni (−0.835). PC2 retained 18.799% of the total variation, and was positively associated with γ- and δ-tocopherol and proteins (0.804, 0.757, and 0.736, respectively) and negatively associated with dietary fiber and carbohydrates (−0.673 and −0.638, respectively). Figure 1 shows the score and loading plot for the aforementioned PCs.

Although these two components retained approximately only about 56% of the total variance, a significant degree of classification is possible. The rice and coconut and coconut drinks had the most negative PC1 and PC2 scores and were grouped together because of their high SFA content, along with myristic acid, due to the presence of coconut. The clustering of such PBMs was also influenced by lower levels of α-tocopherol and higher levels of Ni. The buckwheat, walnut, and soy drinks were characterized by positive PC2 scores. The significantly higher Ca content revealed in buckwheat and in the fortified walnut PBMs was responsible for their clustering along the diagonal of quadrant II, whereas the soy drink, which had higher levels of γ- and δ-tocopherols than the α- isomer, was adjacent to the top of the PC2 axis. All other PBMs (i.e., rice, oat, millet, sorghum, spelt, almond, and rice and hazelnut) were placed in the right area of the plot, corresponding to the most positive PC1 scores. Particularly, almond PBM had the highest PC2 values due to the highest sum of α-, γ-, and δ-tocopherols, whereas those based on rice had the lowest PC2 scores due to the highest concentrations of carbohydrates, fiber, and palmitic acid, as well as lower Ca and Cu contents. In the middle, the remaining PBMs (i.e., oat, sorghum, spelt, millet, and rice and hazelnut) overlapped with each other.

**Figure 2:**
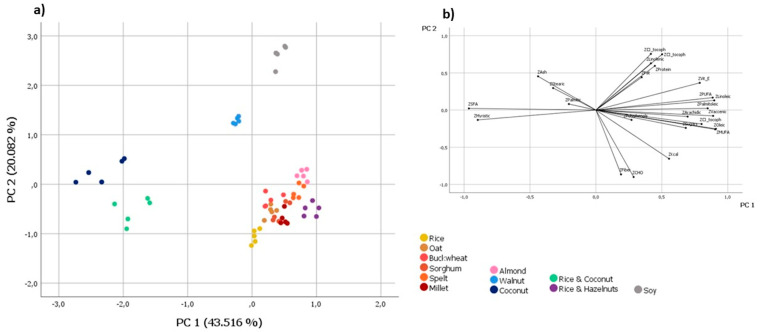
Score (**a**) and loading (**b**) plots of PC2 vs. PC1, with the samples categorized according to the type of PBM, obtained by not considering the inorganic elements among the investigated variables.

A further PCA was performed by removing the inorganic elements from the investigated variables. The new correlation matrix was also factored and suitable for analysis (determinant value was 3.81 × 10^−26^; KMO value was 0.765; Bartlett’s sphericity test had significance at 0.0001). Using the Kaiser criterion, five principal components with eigenvalues greater than one were extracted. These explained 96.995% of the total variance (43.516%, 20.082%, 11.011%, 7.113%, and 5.234%, respectively). The highest positive correlations with PC1 were observed for MUFA (0.910), oleic acid (0.904), cis-vaccenic acid (C18:1 n-7) (0.889), linoleic acid (0.897), PUFA (0.885), and α-tocopherol (0.801), while negative correlations were found for SFA (−0.964) and myristic acid (−0.896). The dominant variables in the second component were δ-tocopherol (0.756), γ-tocopherol (0.751), carbohydrates (−0.900), and dietary fiber (−0.865). The score and loading plots of PC2 vs. PC1 are shown in Figure 2. A better clustering of the investigated samples was achieved in relation to the type of PBM (i.e., cereal-pseudocereal, nut, or legume) due to the shift of samples from buckwheat PBMs. Indeed, they were no longer grouped together with the walnut PBM, which inevitably clustered alone in the component space, but overlapped with the cereal-pseudocereal PBMs. However, the almond and rice and hazelnut PBMs still were within this cluster due to the fact that the contents of total MUFA and cis-vaccenic (C18:1 n-7) and oleic acids were very close to those of cereal and pseudocereal PBMs, such as rice and oat.

### 3.6. Nutritional Value of PBMs

Based on the experimental data, the daily consumption of a cup (~200 mL) of PBMs can effectively contribute to covering the daily reference intake (DRI) of α-tocopherol and/or inorganic elements in an adult consumer, as established by Regulation (EU) No. 1169/2011 [23].

According to the data reported in Table 6, the almond, rice and hazelnut, and soy PBMs may cover more 18–20% of the DRI of α-tocopherol. On the other hand, the rice, coconut, and rice and coconut PBMs were shown to provide 0.88–2% of the DRI, due to the intrinsic low content of this vitamin in raw materials such as rice and coconut.

Considering elements, the buckwheat, coconut, and rice and coconut PBMs are the products that may guarantee the highest contributions of major and trace elements (Table 6). Interestingly, buckwheat PBM may cover ~21% of the DRI of K, and ~37% of the daily requirements of Cu and Se. Coconut and rice and coconut drinks can contribute to ~35% of the daily requirement of Mn and ~31% of the daily requirement of Cu, respectively. Thanks to fortification with calcium phosphate, the walnut PBM can supply up to 35% of the Ca requirement. Conversely, the lowest mineral intakes may come from rice and oat PBMs.

## 4. Conclusions

The comparison of commercial and experimentally determined nutrition labels pointed out that nutrition facts are given to consumers inaccurately, especially with respect to energy, fat, and saturated fat. Also, several imprecisions were found in the declared carbohydrates and proteins. However, the nutritional claims were in line with the EU legislative framework.

Commercial PBMs exhibited such a variability in their chemical compositions that the nutritional standards for a given PBM cannot be unequivocally determined. This is due to the type of plant ingredient, product formulation (i.e., presence/absence of vegetable oil and potential fortification with minerals and/or vitamins), and processing (i.e., wet or dry process). Based on the obtained results, the cereal- and pseudocereal-based PBMs were good sources of MUFA and PUFA, providing many beneficial effects on health, as demonstrated by the evaluated nutritional indices of fat. On the other hand, almond, soy, and rice and hazelnut PBMs were shown to be valid sources of tocopherols, while buckwheat- and coconut-based PBMs showed precious contents of K, Mg, and trace essential elements. As a result, the regular consumption of such PBMs may assure good coverages of DRI of α-tocopherol and various elements, varying from 15% up to 50%, for adult consumers. On the other hand, coconut-based PBMs displayed the worst FA profiles and the lowest α-tocopherol contents, while pseudocereal PBMs, such as rice, oat, sorghum, spelt, and millet, had low mineral contents along with relative scarce daily intakes. The PCA substantially confirmed the nutritional variability of such milk alternatives, indicating that, among the investigated variables, the inorganic elements had more weight in terms of the differentiation of samples based on the PBM type (i.e., (pseudo)cereal, nut, or legume).

This study evaluated, for the first time, the accuracy of the nutrition labels of an array of PBMs available on the Italian market and deepened certain aspects of their chemical composition and nutritional quality. However, several limitations could be pointed out. For example, most of the selected PBM brands were Italian (85.7%). Although the study provides a good representation of national PBM production, it only marginally addressed foreign PBM brands. In the same way, only organic PBMs were taken into account, leaving out the non-organic production that is nevertheless present on the Italian market.

From this point of view, a greater number of brands and types of PBMs certainly reinforce the idea that, unlike cow milk, it is not possible to standardize the composition and nutritional value of these drinks. Additionally, a proper comparison between Italian and “foreign” PBMs could have provided insights into the main trends of the PBM industry in the European context in terms of ingredients, formulation, and processing.

Another drawback was the lack of sensory evaluation and consumer interviews, which would have integrated well with the nutritional and compositional data from this study and, thus, helped to better delineate the scenario of the Italian PBM market.

Considering the growing consumer interest and marketing of PBMs, the present study encourages the future assessment of nutritional quality of further commercial PBMs due to their intrinsic variability, and stresses the need for greater attention and accuracy when developing nutritional labels in order to reliably guide consumers in their dietary choices.

## Figures and Tables

**Table 1:** Description of PBMs under study in terms of ingredient list, nutrition facts, and nutrition claim. In red are the declared values that were found to be out of the tolerance range when compared with the corresponding experimental values (see Table 2). SFA: saturated fatty acids; CHO: carbohydrates.

PBM	Ingredients	Nutrition Facts Label							Nutrition Claim
		Kcal/100 mL	Fat% (SFA%)	CHO% (Sugars%)	Fiber%	Protein%	Salt%	Minerals/Vitamins (mg/100 mL)	
**Tolerance range for nutrient values declared on a label**		To nearest 1 Kcal	≥10 g per 100 mL: to nearest 1 g <10 g and >0.5 g per 100 mL: to nearest 0.1 g ≤0.5 g per 100 mL: 0 g or ≤0.5 g				To nearest 0.01 g	Max. + 45% of the upper bound value Min. − 20% of the lower bound value	
**Rice**	Water, rice (17%), rice oil, salt	56	0.8 (0.2)	12.0 (9.1)	0.0	0.3	0.2	-	With no added sugar
**Oat**	Water, oat (14%), sunflower oil, salt	61	2.0 (0.3)	10.0 (3.6)	<0.5	<0.5	0.13	-	With no added sugar
**Buckwheat**	Water, buckwheat (15%), cold-pressed sunflower seed oil, rice syrup, sea salt	47	1.0 (0.1)	8.0 (4.0)	-	1.0	0.08	-	-
**Sorghum**	Water, sorghum (15%), cold-pressed sunflower seed oil, sea salt	51	1.0 (0.1)	10.0 (3.5)	<0.5	0.5	0.08	-	With no added sugar
**Spelt**	Water, spelt (16%), cold-pressed sunflower seed oil, sea salt	57	1.1 (0.1)	10.5 (5.5)	0.9	0.8	0.08	-	With no added sugar
**Millet**	Water, millet (16%), cold-pressed sunflower seed oil, sea salt	55	1.1 (0.1)	10.5 (5.5)	0.4	0.7	0.08	-	With no added sugar
**Almond**	Water, almond paste (4%), rice starch, pea protein	65	3.7 (0.4)	6.5 (4.0)	<0.5	1.3	0.10	-	-
**Walnut**	Water, cane sugar, walnut paste (2%), calcium phosphate, carob seed flour, sea salt, vitamins: B2, B12, D	25	1.3 (0.2)	2.9 (2.8)	0.1	0.3	0.10	Ca: 120 Vit. B2: 0.21 Vit. B12: 0.38 Vit. D: 0.15	-Low fat -Source of calcium and vitamins
**Coconut**	Water, coconut milk (8%), coconut water (4.8%), rice starch, sea salt	19	1.3 (1.1)	1.7 (1.2)	-	0.2	0.1	-	With no added sugar
**Soy**	Water, soy (8%)	39	2.0 (0.3)	1.7 (0.8)	<0.5	3.5	0.10	-	-
**Rice and coconut**	Water, rice (17%), coconut milk (4%), coconut water (2.4%), sea salt	60	0.8 (0.6)	13.0 (6.5)	-	<0.5	0.08	-	-With no added sugar -Low fat
**Rice and hazelnut**	Water, rice (17%), hazelnuts (3%), sea salt	74	2.3 (0.3)	12.5 (5.5)	0.7	0.5	0.09	-	-

**Table 2:** Nutrition labels experimentally determined for the investigated PBMs. Data are expressed as mean ± standard deviation of n = 5 products of the same brand, each analyzed in triplicate. SFA: saturated fatty acids; CHO: carbohydrates. Bold *p*-values are statistically significant (*p* < 0.05).

PBM	Kcal/100 mL	Fat (%)	SFA (%)	CHO (%)	Sugars (%)	Fiber (%)	Protein (%)	Ash (%)	Ca (mg/100 mL)	Salt (%)
**Rice**	69.72 ± 0.53	0.92 ± 0.14	0.22 ± 0.032	15.41 ± 0.22	11.71 ± 0.10	≤0.5	≤0.5	0.13 ± 0.012	-	0.073 ± 0.010
**Oat**	59.04 ± 1.71	1.41 ± 0.32	0.25 ± 0.060	10.50 ± 0.33	3.82 ± 0.13	≤0.5	0.93 ± 0.13	0.22 ± 0.026	-	0.10 ± 0.022
**Buckwheat**	51.82 ± 1.13	0.986 ± 0.37	0.082 ± 0.014	10.55 ± 0.32	5.24 ± 0.11	≤0.5	≤0.5	0.70 ± 0.072	-	0.043 ± 0.028
**Sorghum**	55.34 ± 0.71	1.05 ± 0.25	0.13 ± 0.013	11.09 ± 0.21	3.88 ± 0.075	≤0.5	≤0.5	0.24 ± 0.025	-	0.092 ± 0.023
**Spelt**	61.89 ± 1.16	0.83 ± 0.20	0.16 ± 0.032	12.22 ± 0. 17	6.43 ± 0.071	1.02 ± 0.10	1.05 ± 0.17	0.39 ± 0.034	-	0.090 ± 0.015
**Millet**	59.41 ± 1.24	0.93 ± 0.26	0.071 ± 0.028	12.63 ± 0.25	6.66 ± 0.13	≤0.5	≤0.5	0.25 ± 0.021	-	0.083 ± 0.038
**Almond**	60.86 ± 6.42	3.12 ± 0.66	0.24 ± 0.070	6.64 ± 0.83	4.14 ± 0.57	≤0.5	1.36 ± 0.13	0.37 ± 0.033	-	0.10 ± 0.013
**Walnut**	21.33 ± 1.68	0.91 ± 0.090	0.15 ± 0.022	3.00 ± 0.23	2.91 ± 0.23	≤0.5	≤0.5	0.51 ± 0.043	139.98	0.094 ± 0.031
**Coconut**	17.94 ± 1.14	1.07 ± 0.25	0.73 ± 0.24	1.88 ± 0.25	1.32 ± 0.13	≤0.5	≤0.5	0.56 ± 0.040	-	0.11 ± 0.023
**Soy**	29.97 ± 1.01	1.71 ± 0.23	0.36 ± 0.038	0.93 ± 0. 37	0.40 ± 0.15	≤0.5	2.50 ± 0.25	0.36 ± 0.038	-	0.13 ± 0.025
**Rice & coconut**	59.7 ± 1.7	1.03 ± 0.38	0.72 ± 0.18	12.53 ± 0.34	6.43 ± 0.27	≤0.5	≤0.5	0.53 ± 0.058	-	0.077 ± 0.011
**Rice & hazelnut**	78.1 ± 2.0	2.53 ± 0.42	0.23 ± 0.017	13.24 ± 0.42	5.66 ± 0.23	0.83 ± 0.095	≤0.5	0.22 ± 0.020	-	0.13 ± 0.035
***p*-value**	**<0.001**	**<0.001**	**<0.001**	**<0.001**	**<0.001**	**<0.001**	**<0.001**	**<0.001**	-	0.99

**Table 3:** FA profiles (%, fw) of the PBMs. Data are expressed as mean GC-FID peak area percent ± standard deviation of n = 5 products of the same brand, each analyzed in triplicate. Health indices of PBMs, such as the atherogenicity index (AI), the thrombogenicity index (TI), the hypocholesterolemic/hypercholesterolemic ratio (h/H), and the n-6/n-3 ratio are also reported. SFA: saturated fatty acids; MUFA: monounsaturated fatty acids; PUFA: polyunsaturated fatty acids. Bold *p*-values are statistically significant (*p* < 0.05).

	Rice	Oat	Buckwheat	Sorghum	Spelt	Millet	Almond	Walnut	Coconut	Soy	Rice and Coconut	Rice and Hazelnut	*p*-Value
**C8:0**	nd	nd	nd	nd	nd	nd	nd	nd	10.34 ± 1.92	nd	7.69 ± 1.15	nd	-
**C10:0**	nd	nd	nd	nd	nd	nd	nd	nd	7.57 ± 1.14	nd	6.30 ± 1.17	nd	-
**C12:0**	nd	nd	nd	nd	nd	nd	nd	nd	39.40 ± 4.29	nd	41.70 ± 2.49	nd	-
**C14:0**	0.38 ± 0.043	0.16 ± 0.042	0.12 ± 0.056	0.082 ± 0.021	0.086 ± 0.054	0.030 ± 0.024	0.071 ± 0.032	0.063 ± 0.033	19.36 ± 3.65	0.08 ± 0.031	19.10 ± 3.26	0.13 ± 0.080	**<0.001**
**C16:0**	15.89 ± 1.42	10.60 ± 1.18	7.84 ± 1.12	6.58 ± 1.43	8.02 ± 1.21	6.97 ± 1.06	7.48 ± 1.11	9.84 ± 1.79	8.64 ± 0.99	10.14 ± 1.05	9.17 ± 1.14	6.76 ± 1.05	**<0.001**
**C18:0**	2.29 ± 0.49	3.06 ± 1.08	3.54 ± 1.76	3.81 ± 0.90	3.05 ± 0.96	3.36 ± 0.53	1.68 ± 0.94	4.94 ± 1.01	3.11 ± 0.79	4.88 ± 0.83	3.76 ± 0.66	0.17 ± 0.092	**<0.001**
**C20:0**	1.14 ± 0.18	0.23 ± 0.093	0.33 ± 0.10	0.30 ± 0.077	0.36 ± 0.10	0.46 ± 0.13	0.18 ± 0.056	0.16 ± 0.052	0.060 ± 0.030	0.36 ± 0.11	0.044 ± 0.032	0.20 ± 0.090	**<0.001**
**SFA**	19.70 ± 5.83	14.05 ± 3.95	11.82 ± 3.00	10.76 ± 2.62	11.51 ± 3.02	10.82 ± 2.69	9.41 ± 2.77	14.99 ± 3.85	88.48 ± 13.28	15.47 ±3.93	87.76 ± 14.14	7.27 ± 2.53	**<0.001**
**C16:1n-7**	0.10 ± 0.054	0.15 ± 0.053	0.22 ± 0.084	0.23 ± 0.11	0.19 ± 0.070	0.17 ± 0.082	0.42 ± 0.14	0.10 ± 0.041	0.030 ± 0.022	0.13 ± 0.063	0.020 ± 0.022	0.14 ± 0.061	**<0.001**
**C18:1n-9**	40.34 ± 3.00	58.08 ± 4.68	32.84 ± 3.74	34.82 ± 3.68	30.52 ± 2.83	37.36 ± 5.15	57.25 ± 6.50	17.71 ± 2.08	6.02 ± 1.83	21.33 ± 3.07	6.05 ± 1.19	59.48 ± 7.39	**<0.001**
**C18:1n-7**	1.27 ± 0.38	1.12 ± 0.23	0.98 ± 0.29	0.84 ± 0.39	0.89 ± 0.17	0.98 ± 0.38	0.57 ± 0.14	0.38 ± 0.19	0.040 ± 0.012	1.06 ± 0.27	0.13 ± 0.080	1.41 ± 0.69	**<0.001**
**MUFA**	41.70 ± 22.90	59.35 ± 33.17	34.04 ± 18.61	35.90 ± 19.80	31.60 ± 17.31	38.52 ± 21.24	58.23 ± 32.77	18.19 ± 10.09	6.09 ± 3.46	22.53 ± 11.98	6.20 ± 3.45	61.04 ± 33.90	**<0.001**
**C18:2n-6**	32.97 ± 3.27	21.09 ± 2.31	49.67 ± 5.32	47.84 ± 3.34	51.35 ± 4.63	46.14 ± 4.09	27.94 ± 4.47	51.61 ± 2.54	1.03 ± 0.23	50.09 ± 3.31	2.15 ± 0.93	26.82 ± 3.90	**<0.001**
**C18:3n-3**	1.34 ± 0.57	0.52 ± 0.20	0.30 ± 0.13	0.45 ± 0.25	0.92 ± 0.25	0.38 ± 0.20	0.53 ± 0.24	11.84 ± 1.74	0.072 ± 0.045	7.55 ± 1.20	0.21 ± 0.16	0.29 ± 0.14	**<0.001**
**PUFA**	34.31 ± 22.36	21.61 ± 14.54	49.96 ± 34.91	48.29 ± 33.51	52.27 ± 35.66	46.52 ± 32.36	28.47 ± 19.38	63.46 ± 28.12	1.09 ± 0.68	57.64 ± 30.09	2.36 ± 1.37	27.11 ± 18.76	**<0.001**
**AI**	0.23	0.14	0.10	0.082	0.10	0.083	0.091	0.12	17.48	0.13	14.87	0.083	-
**TI**	0.45	0.33	0.27	0.24	0.25	0.24	0.21	0.21	8.15	0.26	6.53	0.16	-
**h/H**	4.59	7.41	10.40	12.49	10.22	11.98	11.35	8.20	0.11	7.72	0.12	12.55	-
**n-6/n-3**	24.56	40.24	167.80	106.78	55.81	122.72	53.12	4.36	15.55	6.64	10.25	91.84	-

**Table 4:** Contents of α-γ-δ-tocopherols, their sums (mg/L, fw), and total polyphenols (mg GAE/100 mL, fw) in PBMs. Data are expressed in terms of the mean ± standard deviation of n = 5 products of the same brand, each analyzed in triplicate. Bold *p*-values are statistically significant (*p* < 0.05).

	α-Tocopherol	γ-Tocopherol	δ-Tocopherol	Total Tocopherols (α + γ + δ)	Total Polyphenols
**Rice**	0.59 ± 0.21	0.47 ± 0.15	0.13 ± 0.04	1.20 ± 0.29	13.16 ± 1.40
**Oat**	2.07 ± 0.19	0.40 ± 0.12	0.090 ± 0.022	2.14 ± 1.05	18.01 ± 2.20
**Buckwheat**	1.58 ± 0.46	0.52 ± 0.09	0.19 ± 0.10	2.29 ± 0.55	52.27 ± 4.99
**Sorghum**	4.06 ± 0.68	0.59 ± 0.15	0.13 ± 0.07	4.79 ± 0.87	17.70 ± 1.98
**Spelt**	5.66 ± 0.63	1.12 ± 0.30	0.39 ± 0.10	7.17 ± 0.69	34.25 ± 2.90
**Millet**	3.12 ± 0.73	0.54 ± 0.17	0.18 ± 0.05	3.84 ± 0.72	16.25 ± 2.19
**Almond**	12.30 ± 2.70	1.09 ± 0.34	0.28 ± 0.19	13.68 ± 2.88	13.83 ± 1.76
**Walnut**	1.69 ± 0.29	1.10 ± 0.20	0.65 ± 0.22	3.44 ± 0.59	17.49 ± 2.54
**Coconut**	0.19 ± 0.10	0.30 ± 0.13	0.041 ± 0.030	0.53 ± 0.25	5.40 ± 0.79
**Soy**	0.66 ± 0.21	8.67 ± 1.04	2.85 ± 1.02	12.19 ± 2.03	20.61 ± 2.32
**Rice and coconut**	0.55 ± 0.19	0.38 ± 0.12	0.16 ± 0.073	1.09 ± 0.33	9.54 ± 1.61
**Rice and hazelnut**	9.28 ± 1.58	1.80 ± 0.39	0.21 ± 0.074	11.29 ± 1.99	13.58 ± 1.64
***p*-value**	**<0.001**	**<0.001**	**<0.001**	**<0.001**	**<0.001**

**Table 5:** Element profiles (mg/L, fw) of PBMs. For every PBM, data are expressed as the mean ± sd of n = 5 products of the same brand, each analyzed in triplicate. The limit of quantification (LOQ) of Se was 0.002 mg/L; that of Cr was 0.004 mg/L; and those of As, Cd, and Pb were 0.003 mg/L. Bold *p*-values are statistically significant (*p* < 0.05).

	Rice	Oat	Buckwheat	Sorghum	Spelt	Millet	Almond	Walnut	Coconut	Soy	Rice and Coconut	Rice and Hazelnut	*p*-Value
**Na**	265.51 ± 28.64	397.76 ± 36.60	178.20 ± 38.47	344.74 ± 16.98	340.34 ± 40.12	337.31 ± 23.73	420.32 ± 29.70	360.55 ± 29.82	351.16 ± 34.09	254.17 ± 33.96	239.58 ± 17.89	486.56 ± 60.72	**<0.001**
**K**	237.51 ± 38.19	424.80 ± 49.65	2057.81 ± 189.23	579.68 ± 54.00	609.83 ± 49.07	564.19 ± 52.38	344.01 ± 24.83	251.40 ± 35.70	1544.67 ± 57.39	1020.31 ± 81.21	1706.33 ± 115.28	484.37 ± 75.45	**<0.001**
**Mg**	53.09 ± 11.37	73.17 ± 20.17	278.26 ± 26.22	62.28 ± 23.02	50.68 ± 14.72	59.30 ± 21.78	141.63 ± 24.17	91.20 ± 24.37	177.81 ± 61.59	156.02 ± 19.11	181.56 ± 30.62	122.75 ± 23.35	**<0.001**
**Ca**	119.44 ± 23.65	207.47 ± 22.64	769.53 ± 45.50	160.65 ± 50.88	151.51 ± 33.14	157.92 ± 39.32	503.31 ± 22.81	1399.84 ± 35.26	616.72 ± 42.68	441.76 ± 30.91	296.32 ± 25.93	261.31 ± 46.64	**<0.001**
**Fe**	0.50 ± 0.24	0.62 ± 0.24	5.12 ± 1.69	0.88 ± 0.28	0.78 ± 0.39	0.75 ± 0.31	2.61 ± 0.86	2.97 ± 0.98	4.34 ± 1.67	4.58 ± 1.26	5.36 ± 1.84	2.42 ± 0.60	**<0.001**
**Mn**	1.02 ± 0.37	0.84 ± 0.33	2.84 ± 1.14	0.83 ± 0.28	0.67 ± 0.15	0.61 ± 0.35	0.97 ± 0.26	0.84 ± 0.42	3.48 ± 1.32	1.80 ± 0.53	2.94 ± 0.98	1.67 ± 0.39	**<0.001**
**Cu**	0.08 ± 0.03	0.21 ± 0.12	1.85 ± 1.19	0.42 ± 0.35	0.44 ± 0.40	0.36 ± 0.31	0.74 ± 0.35	0.70 ± 0.30	0.95 ± 0.36	2.49 ± 0.91	1.55 ± 0.55	1.22 ± 0.73	**<0.001**
**Zn**	0.64 ± 0.19	0.77 ± 0.40	5.85 ± 1.22	0.77 ± 0.30	1.07 ± 0.48	1.17 ± 0.58	1.82 ± 0.65	1.59 ± 0.55	2.47 ± 0.82	2.87 ± 0.80	2.67 ± 1.09	1.29 ± 0.40	**<0.001**
**Se**	<LOQ	<LOQ	0.10 ± 0.037	0.016 ± 0.010	<LOQ	0.014 ± 0.0071	0.0093 ± 0.0052	0.016 ± 0.0052	0.044 ± 0.029	0.0061 ± 0.0034	0.037 ± 0.017	<LOQ	**<0.001**
**Ni**	0.077 ± 0.052	0.092 ± 0.035	0.49 ± 0.25	0.056 ± 0.034	0.065 ± 0.045	0.072 ± 0.038	0.043 ± 0.027	0.18 ± 0.053	0.54 ± 0.25	0.16 ± 0.075	0.59 ± 0.25	0.057 ± 0.025	**<0.001**
**Cr**	0.0092 ± 0.0051	<LOQ	<LOQ	<LOQ	<LOQ	<LOQ	<LOQ	0.0053 ± 0.0032	0.027 ± 0.013	0.012 ± 0.0080	0.020 ± 0.013	<LOQ	-
**As**	0.028 ± 0.012	0.0062 ± 0.0021	0.013 ± 0.0083	<LOQ	<LOQ	<LOQ	<LOQ	0.0091 ± 0.0062	0.0081 ± 0.0032	<LOQ	0.0043 ± 0.0010	0.018 ± 0.0072	**<0.001**
**Cd**	<LOQ	<LOQ	0.013 ± 0.0042	<LOQ	<LOQ	<LOQ	<LOQ	<LOQ	0.0053 ± 0.0021	0.0042 ± 0.0020	<LOQ	<LOQ	-
**Pb**	0.0072 ± 0.0033	0.0051 ± 0.0010	0.017 ± 0.0085	0.0064 ± 0.0025	<LOQ	<LOQ	0.0053 ± 0.0013	0.011 ± 0.0037	0.0060 ± 0.0034	0.0052 ± 0.0022	0.010 ± 0.0046	0.0059 ± 0.0013	**<0.001**

**Table 6:** Daily intake (%) of certain nutrients derived from the daily consumption of 200 mL of PBMs. Data were calculated with respect to the daily reference intake (DRI *) of an adult consumer, as referenced by the Reg. (UE) 1169/2011.

	Rice	Oat	Buckwheat	Sorghum	Spelt	Millet	Almond	Walnut	Coconut	Soy	Rice and Coconut	Rice and Hazelnut
**A-tocopherol**	2.00	3.57	3.82	7.98	11.95	6.40	22.80	5.74	0.88	20.31	1.81	18.82
**K**	2.38	4.25	20.58	5.80	6.10	5.64	3.44	2.51	15.45	10.20	17.06	4.84
**Mg**	2.83	3.90	14.84	3.32	2.70	3.16	7.55	4.86	9.48	8.32	9.68	6.55
**Ca**	2.99	5.19	19.24	4.02	3.79	3.95	12.58	35.00	15.42	11.044	7.41	6.53
**Fe**	0.71	0.89	7.31	1.26	1.11	1.07	3.72	4.24	6.19	6.54	7.66	3.45
**Mn**	10.18	8.39	28.44	8.26	6.72	6.08	9.73	8.41	34.83	18.00	29.42	16.68
**Cu**	1.57	4.19	37.05	8.39	8.80	7.11	14.76	13.98	18.96	49.78	30.92	24.45
**Zn**	1.27	1.54	11.70	1.54	2.14	2.34	3.65	3.18	4.94	5.75	5.33	2.58
**Se**	0.00	0.00	37.60	5.89	0.00	5.09	3.27	5.75	15.93	2.25	13.53	0.00

* DRIs of α-tocopherol: 12 mg; K: 2000 mg; Mg: 375 mg; Ca: 800 mg; Fe: 14 mg; Mn: 2 mg; Cu: 1 mg; Zn: 10 mg; Se: 55 µg.

## Data Availability

Data is contained within the article.
